# Survival dynamics of stick insect and the impact of environmental factors on natural fungal infection during the rainy season

**DOI:** 10.3389/fmicb.2024.1383055

**Published:** 2024-04-24

**Authors:** Donggyu Min, Soobin Shin, Noh-Hyun Lee, Min Jeong Baek, Sun-Jae Park, Kwang-Hyung Kim, Hokyoung Son, Jong-Kook Jung

**Affiliations:** ^1^Department of Agricultural Biotechnology, Seoul National University, Seoul, Republic of Korea; ^2^Department of Forest Environment Protection, Kangwon National University, Chuncheon, Republic of Korea; ^3^National Institute of Biological Resources, Incheon, Republic of Korea; ^4^Research Institute of Agriculture and Life Sciences, Seoul National University, Seoul, Republic of Korea

**Keywords:** *Ramulus mikado*, *Metarhizium phasmatodeae*, rainy season, biocontrol agent, entomopathogenic fungi

## Abstract

Phasmatodea, commonly known as stick insects, are recognized as noteworthy pests globally, impacting agriculture and forest ecosystems. Among them, the outbreak of *Ramulus mikado* has emerged as a notable concern in East Asian forests. Recently, *Metarhizium phasmatodeae* has been identified as utilizing stick insects as hosts. We have observed evidence of this entomopathogenic fungus infecting stick insects. Given the increase in these occurrences during the rainy period, this study investigated the relationship between the survival of *R. mikado* and the *M. phasmatodeae* infection during the rainy seasons of 2022 and 2023. We collected stick insects in two representative forests of the Republic of Korea and examined insect survival, fungal infection, and various environmental factors. No infections were detected in specimens collected in June before the rainy season, but from July onwards, both the mortality of *R. mikado* and the fungal infection substantially increased. By the last sampling date of each year, 75% (2022), 71.4% (2023) of the specimens were infected, and over 90% of the total individuals succumbed as a result. Fungi isolated from deceased *R. mikado* were successfully identified as *M. phasmatodeae* using morphological and taxonomic approaches. Various statistical analyses, including principal component analysis and modeling, revealed a robust association between fungal infection and the survival of stick insects. The results highlight the correlation between mass deaths of stick insects and fungal infection, particularly during the summer rainy season. These findings offer valuable insights for forecasting *R. mikado* population in the upcoming year and developing effective pest control strategies.

## 1 Introduction

Stick insect, *Ramulus mikado* (Phasmatodea: Phasmatidae), is a wingless and parthenogenetic forest pest. *R. mikado* follows a univoltine reproductive cycle with obligatory embryonic diapause. Eggs are primarily laid in summer, and individuals overwinter as pharate first-instar nymphs (Yamaguchi and Nakamura, 2015). The stick insects pose a significant threat by causing serious defoliation in various deciduous trees. They exhibit polyphagous behavior by feeding on leaves. The long-term and repetitive defoliation caused by a high stick insect population can lead to premature seedling death and a reduction in the diameter growth of trees (Mazanec, 1967, 1968; Liu, 2021). In 2020, an outbreak of the stick insect in the urban forests near the metropolitan area in the Republic of Korea caused damage to 19 ha, escalating to 158 ha in 2021 and 981 ha in 2022 (Korean Forest Service, 2023). A comparable situation occurred in Japan, where *R. mikado* led an outbreak near residential areas over 2–3 years (Yano et al., 2021). Considering the economic and quality risks associated with the pest outbreak, various control methods, encompassing physical, chemical, and biological approaches, have been proposed (Ji et al., 2011; Klapwijk et al., 2016). Typically, synthetic pesticides such as fenitrothion and ethofenprox are applied to control *R. mikado* (Korean Forest Service, 2023). However, this method can affect non-target insects living in fields, leading to issues like bioaccumulation and the risk of side effects (Blacquière et al., 2012; Rahman et al., 2020; Yan et al., 2020).

Entomopathogenic fungi have emerged as highly promising alternative biocontrol agents to chemical insecticides (Erler and Ates, 2015; Perumal et al., 2023; Vivekanandhan et al., 2023; Perumal et al., 2024a,b). *Metarhizium* and *Beauveria* species are two main generalist entomopathogenic fungi widely employed in pest control across various insect species, including agricultural pests (Mascarin and Jaronski, 2016; Peng et al., 2021; Krutmuang et al., 2023; Vivekanandhan et al., 2024; Perumal et al., 2024a,b). Notably, the fungi of the genus *Metarhizium* have been recognized for their ability to infect and eliminate a broad range of arthropods (Zimmermann, 2007; Brunner-Mendoza et al., 2019; Vivekanandhan et al., 2022). This process involves the release of cuticle-degrading enzymes and the application of mechanical pressure exerted by the appressorium (Leger et al., 1986; Barelli et al., 2016). Upon successful penetration of the host insect, asexual spores (blastospores) are produced to facilitate dispersal in the insect hemocoel. Throughout the invasion of the insect body, depletion of nutrients in the hemocoel and the fat body occurs, eventually leading to the death of the host (Gao et al., 2011; Peng et al., 2022). In the case of the order Phasmatodea (Insecta), *M. phasmatodeae* has recently been reported as a novel species, utilizing stick insects as its hosts (Mongkolsamrit et al., 2020; Thanakitpipattana et al., 2020). In domestic cases, there have been reports of *R. mikado* being infected by *M. anisopliae*, and its biocontrol has been confirmed through artificial inoculation (Kim et al., 2010; Jung et al., 2023).

Before applying cultured entomopathogenic fungi to the field, it is necessary to conduct preliminary research on how these biocontrol agents react to environmental conditions during host infection. Their biocontrol activities may vary under actual field conditions compared to laboratory environments during inoculation (De La Rosa et al., 2000; Stafford and Allan, 2014). Particularly, the subtropical frontal zone of East Asia experiences heavy summer rainfall (Choi et al., 2020). The Korean rainy season, known as Changma, is characterized by a prominent increase in rainfall from late June to August, leading to elevated temperatures and relative humidity (Wang et al., 2007). Studies suggest that, entomopathogenic fungi, including the genus *Metarhizium* tend to enhance their virulence in response to increased temperature and humidity (Arthurs and Thomas, 2001; Goble et al., 2016). The genus *Metarhizium* is recognized for its presence in soil environments and its infection of insects inhabiting these areas (Mcguire and Northfield, 2020; Tamayo-Sánchez et al., 2022). However, research on *Metarhizium* species infections in stick insects, predominantly residing in the canopy layer throughout most of their life cycle, is limited. Taken together, we hypothesized that the survival of *R. mikado* could be affected by infection with entomopathogenic fungi, particularly with enhanced insecticidal performance during the rainy season.

The objectives of this study were 1) to assess the survival of *R. mikado* and the impact of *M. phasmatodeae* infection during the rainy season and 2) to explore the environmental factors affecting the biocontrol activity of *M. phasmatodeae*. Our investigation revealed that the survival of *R. mikado* is influenced by entomopathogenic fungi in the forest ecosystem, along with other environmental factors. We also observed a notable increase in the natural infection of *M. phasmatodeae* during the summer rainy season. These findings provide valuable insights for predicting the population dynamics of *R. mikado* in the upcoming years and for devising effective pest control strategies.

## 2 Materials and methods

### 2.1 Sample collection

Stick insects were identified morphologically by using taxonomic keys from National Institute of Biological Resources.^1^ Nymphs and adults of *R. mikado* were collected from Mt. Cheonggye (N 37° 24′ 21″ E 127° 00′ 02″) and Mt. Geumam (N 37° 30′ 42″ E 127° 10′ 52″) in Gyeonggi Province in 2022 and 2023, respectively. In each area, sampling was conducted by hand-picking and beating vegetation. The sampling was performed four times in 2022 and six times in 2023.

Approximately 40 to 50 individuals were collected per sampling, and 10 individuals were reared in cages (30 cm × 30 cm × 30 cm) at room temperature (RT) to minimize spatial stress. Specimens were chosen randomly and placed in separate cages for rearing. Cherry and oak branches with leaves (*Prunus sargentii* and *Quercus mongolica*) were supplied as food sources. Dead individuals were separated and individually transferred to petri dishes (90 × 15 mm) containing moistened filter paper to promote fungal sporulation. The dishes were incubated at 25 ± 1°C in darkness.

### 2.2 Data investigation

To evaluate survival and infection rates, dead individuals were counted every day and incubated to observe fungal conidia. Survival and infection rates were calculated as follows:


$$\text{Survival}\;\text{rate}\;\left(\%\right)=\frac{\text{Number}\;\text{of}\;\text{dead}\;\text{samples}}{\text{Number}\;\text{of}\;\text{collected}\;\text{samples}}\times 100$$

$$\text{Infection}\;\text{rate}\;\left(\%\right)=\frac{\text{Number}\;\text{of}\;\text{infected}\;\text{dead}\;\text{samples}}{\text{Number}\;\text{of}\;\text{collected}\;\text{samples}}\times 100$$

To obtain environmental information, data loggers, HOBO Pro v2 Temp / RH (Onset, Seoul, Republic of Korea) were mounted on trees for each sampling site and set to record temperature and relative humidity every hour. And then they were represented as average values using data from the 14 days preceding the sampling date. For precipitation, daily data obtained from the nearby Automatic Weather Station (AWS, https://data.kma.go.kr) at the sites were represented as cumulative precipitation data over the same period.

### 2.3 Fungal isolation

The strains used in this study were isolated from deceased *R. mikado* specimens were collected in the forest. We collected stick insects ranging from the 5th instar nymphs to adults. We randomly selected insects per sampling site and cultured fungi on potato dextrose agar (PDA: Difco, Detroit, United States) plates (90 × 15 mm) containing 100 μg/mL of penicillin and 100 μg/mL of streptomycin. The plates were sealed with parafilm and incubated at 25 ± 1°C in darkness. The emergence of mycelia was monitored daily, and the fungus was reisolated by single spore isolation to obtain a pure fungal culture. All pure isolates were stored in 20% glycerol at −80°C until use.

### 2.4 Morphological observation

To assess macro- and micro-morphological characters, each fungal isolate was grown on PDA at 25 ± 1°C for 14 days. The fungal spores were also obtained from PDA plates cultured for 14 days. Specimens were observed and photographed at 400 × magnification in a DM6 B microscope (Leica Microsystems, Wetzlar, Germany) equipped with a Leica DMC6200 camera.

### 2.5 Molecular identification and phylogenetic analyses

Fungal genomic DNA was extracted following the standard protocol (Specht et al., 1982) with minor modifications. Briefly, a 7-day-old fungal mass was placed in a 1.5 mL tube containing a mini plastic pestle after adding 450 μL of grinding buffer (20 mM Na_4_EDTA; 0.1 M Tris–HCl, pH 7.5; 1.4 M NaCl). Ground mycelia were mixed by inversion and centrifuged at 13,000 rpm for 10 min. The supernatant was transferred to a new tube, and the same volumes of isopropanol were added. Nucleic acids were precipitated by centrifugation at 13,000 rpm for 10 min at 4°C, dried for 15 min, and resuspended in 20 μL of distilled water. The extracted DNA was preserved at −20°C and used as a template for PCR amplifications. As previously described (White et al., 1990), PCR amplifications were carried out in 50 μL reactions using Taq DNA polymerase (Takara Shuzo, Japan), 20 pmol of each primer, and roughly 50 ng of template DNA for four loci of all strains; the internal transcribed spacer (ITS) region of 18 S-28 S nuclear ribosomal DNA, and 5′ intron-rich region translation elongation factor 1-α (5’*tef*). PCR primer pairs used to amplify the gene regions for this study were: ITS1 (GARTGYCCDGGDCAYTTYGG)/ITS4 (CCNGCDATNTCRTTRTCCATRTA) for ITS (White et al., 1990), EF1T (ATGGGTAAGGARGACAAGAC)/EF2T (GGAAGTACCAGTGATCATGTT) for 5’*tef* (Bischoff et al., 2006). Thermocycler conditions for amplification of the DNA regions followed previously described protocols (Rehner and Buckley, 2005). Amplified PCR products were purified using the MEGAquick-spinTM plus Fragment DNA Purification kit (Intron, Seongnam, Republic of Korea) and sequenced (Bioneer, Daejeon, Republic of Korea).

Analysis of DNA sequences was performed with Seqman Pro (DNAStar, Madison, WI, United States) to assemble and edit forward and reverse sequences. These sequences were aligned using Clustal W by MEGA-X (Kumar et al., 2018). Sequences of ITS and 5’*tef* from related species were retrieved from the NCBI Genebank to elucidate relationships in *Metarhizium* (Mongkolsamrit et al., 2020). Maximum likelihood (ML) analyses were performed with the general time reversible model with Invariant sites and Gamma distribution (GTR + I + G) with 1,000 bootstrap replicates using RAxML (Edler et al., 2021). This model was selected due to its comprehensive approach, accounting for base substitution rates, invariant sites, and rate variations among sites (Abadi et al., 2019). Bayesian posterior probabilities (BPP) were performed using Markov Chain Monte Carlo sampling (MCMC) in MrBayes v. 3.2.7 (Ronquist et al., 2012). Six simultaneous Markov Chains were run for 100,000 generations, and trees were sampled every 1,000th generation. The phylogenetic trees were visualized in Fig Tree v. 1.4.4 (Rambaut, 2009). The NCBI accession numbers of the three strains in this study are listed in Supplementary Table S2.

### 2.6 Insect bioassay

To verify the pathogenicity of *M. phasmatodeae* against *R. mikado*, adult *R. mikado* specimens were used in the bioassay as described previously (Kim et al., 2020). Three fungal isolates were randomly selected and cultured on PDA plates (90 × 15 mm) at 25 ± 1°C for 14 days. To induce exposure to conidia, individual adult *R. mikado* specimen was placed on the fungal culture. After 1 h, the infected adult was transferred to a plastic cup (20 mm × 90 mm × 81.6 mm) along with a fresh cherry branch (*Prunus sargentii*) as a food source. The stick insects were reared in the cups at 25 ± 1°C for 14 days, with one milliliter of sterile distilled water supplied every 2 days to maintain high humidity. The number of deceased adults was recorded daily. Once stick insects died, they were moved to petri dishes (90 × 15 mm) with moistened filter paper to assess infection by *M. phasmatodeae*. This bioassay was repeated three times.

### 2.7 Statistical analysis

The survival of *R. mikado* post-sampling was visualized using the Kaplan–Meier curve (Kaplan and Meier, 1958). Any insects surviving beyond this time were regarded as “censored.” Log-rank (Mantel-Cox) pair-wise comparisons were used to evaluate the difference in survivorship based on the sampling dates.

Additionally, principal component analysis (PCA) was used to determine the multiple correlations based on the sampling dates (Wold et al., 1987). In the PCA biplot, survival and infection rates, and environmental factors were represented by vectors, and sample IDs were represented by symbols. Angles between vectors reflect their correlation, which can be estimated as the cosine of the angle between any two vectors; vectors pointing in the same direction have a positive correlation, those pointing in opposite directions are negatively correlated, and vectors at right angles mean that vectors are not correlated.

The relationships between survival rate and each of the variables (infection rate, temperature, relative humidity, and precipitation) were investigated. In this study, nonlinear or linear models that accurately describe these relationships were fitted using the R packages nls (nonlinear model) or lm (linear model). The performance of each model was estimated using the coefficient of determination (R^2^) based on the observed value and the predicted value from the model. All data analyses were performed using the software R 4.3.2 and SPSS 25.

## 3 Results

### 3.1 Survey on the survival of *R. mikado* in the summers of 2022 and 2023

**Figure 1:**
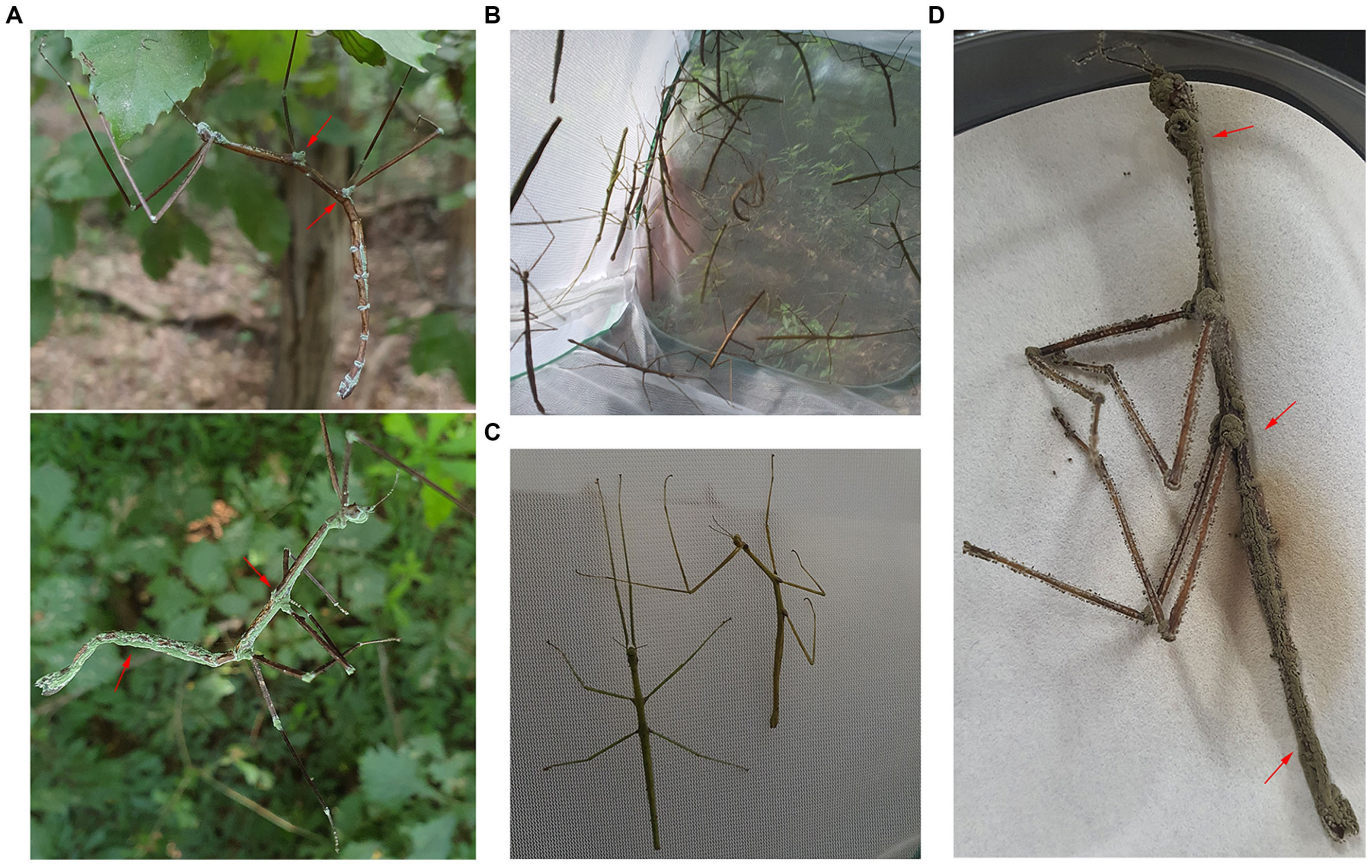
Disease symptoms of *Ramulus mikado*. **(A)** *R. mikado* infested on the body surface presenting a symptom of paralysis. Green spores (red arrows) were formed on the carcass of *R. mikado*. **(B)** Collected *R. mikado* samples. **(C)** Live (left) and dead (right) adults of *R. mikado* were observed. **(D)** Fungal spore masses covered the carcasses of *R. mikado* after incubation in humid conditions.

Diseased stick insects exhibiting paralysis-like symptoms and green spore formation on their body surfaces were observed in some mountains of the Republic of Korea (Figure 1A). Some of these infected stick insects were found deceased, with a notable prevalence during the summer season. Therefore, we aimed to monitor the survival of these insects over time and uncover potential causes of mortality during specific periods.

To assess the survival of *R. mikado*, we collected a total of 461 individuals through four samplings in Mt. Cheonggye (CM) and six samplings in Mt. Geumam (GM) in the summers of 2022 and 2023, respectively (Figures 1B,C; Table 1). The collected individuals underwent a two-week rearing process, revealing that dead insects were covered by fungal spore masses (Figure 1D). In the CM group, the survival rates for CM 3 and CM 4 were 2.7% and 7.5%, respectively, by day 14. In instances where spore masses were detected in dead insects, we classified them as “infected” and investigated infection rates among the deceased. Notably, infection rates exceeded 50% for both the CM and GM groups. In the GM group, survival rates for GM 5 and GM 6 were less than 50%, with fungal infection initiating at GM 3 and gradually escalated, reaching a peak infection rate of 70% by GM 6.

**Table 1:** Information of sampling location.

Date	Sample ID	GPS coordinate	Location	Province	Number of collected insects
10 Jun 2022	CM 1	37°24′21”N 127°00′02″E	Mt. Cheonggye	Gyeonggi	30
24 Jun 2022	CM 2				40
9 Jul 2022	CM 3				37
22 Jul 2022	CM 4				40
9 May 2023	GM 1	37°30′42”N 127°10′52″E	Mt. Geumam	Gyeonggi	50
2 Jun 2023	GM 2				53
16 Jun 2023	GM 3				50
1 Jul 2023	GM 4				50
21 Jul 2023	GM 5				55
3 Aug 2023	GM 6				56

Nymphs and adults *R. mikado* were sampled from Mt. Cheonggye and Mt. Geumam in 2022 and 2023, respectively. The sampling was conducted four times in 2022 and six times in 2023. Approximately 40 to 50 individuals of insects were collected per sampling.

**Figure 2:**
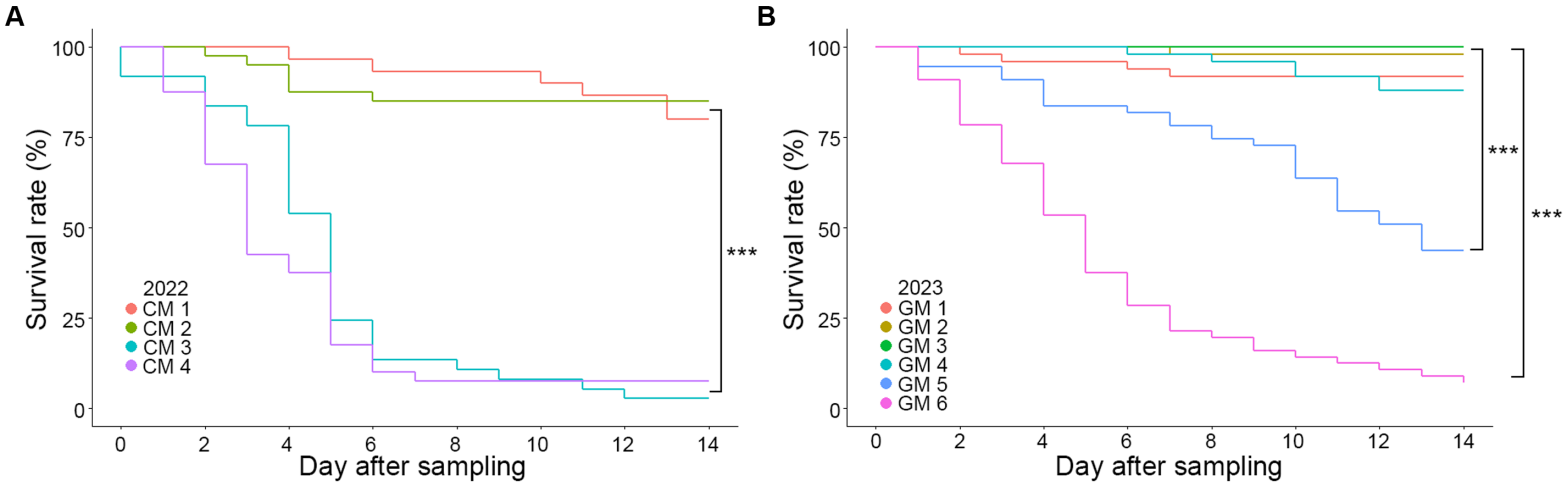
Survival curves according to Kaplan–Meier analysis of *R. mikado*. Survival rates were analyzed for *R. mikado* samples which were collected in **(A)** 2022 and **(B)** 2023. Mortality was examined daily by counting the number of dead insects for 14 days. Asterisk indicates a significant difference in survival among the groups (*p* < 0.001, log-rank test).

A comparison of survival rates based on the sampling dates (CM 1–4 and GM 1–6 groups) indicated a significant decrease in survival rates as the sampling date progressed for both CM and GM groups (Figure 2). Log-rank pair-wise comparisons of survival curves were conducted to assess the statistical significance, providing the chi-square values (*X*^2^) and significance levels (*p*) (Supplementary Table S3). A statistically significant difference in survival rates was observed depending on the sampling period. As an example, in both 2022 and 2023, the final sampling group (CM 4, GM 6) exhibited a rapid mortality rate compared to the first sampling group (CM 1, GM 1) (*X*^2^_(CM)_ = 48.547, *p* < 0.001; *X*^2^_(GM)_ = 81.694, *p* < 0.001). Additionally, the mortality due to fungal infection varied based on the sampling period, with over 70% of all specimens being infected in the final sampling group over the course of 2 years (Table 2). Collectively, our findings suggest a significant decline in the survival rates of *R. mikado* as the sampling period progresses. This prompts further exploration into whether the mortality of these insects is linked to fungal infection.

**Table 2:** Investigation of the survival and infection rates and environmental factors.

Sample ID	Survival rate (%)	Infection rate (%)	Temperature (°C)	Relative humidity (%)	Precipitation (mm)
CM1	80.0	0.0	19.2	70.8	1.0
CM2	85.0	0.0	20.9	84.0	89.5
CM3	2.7	51.4	24.6	92.5	328.5
CM4	7.5	75.0	23.7	89.7	187.5
GM1	92.0	0.0	13.9	62.8	98.0
GM2	98.1	0.0	19.2	74.2	67.0
GM3	100.0	0.0	19.8	79.8	30.5
GM4	88.0	2.0	22.6	84.9	120.5
GM5	43.6	16.4	23.3	93.8	294.5
GM6	7.1	71.4	25.8	91.1	46.0

Sampling individuals were reared in 30 cm^3^ meshed cages, with 10 individuals in each for 2 weeks to check survivorship. Dead individuals were placed in petri-dishes and incubated at 25°C and the infection of *M. phasmatodeae* was investigated based on spores on carcass. Temperature and relative humidity are represented as average values for 2 weeks by HOBO data loggers. Precipitation represents the total accumulation for 2 weeks by AWS.

### 3.2 Fungal species identification

**Figure 3:**
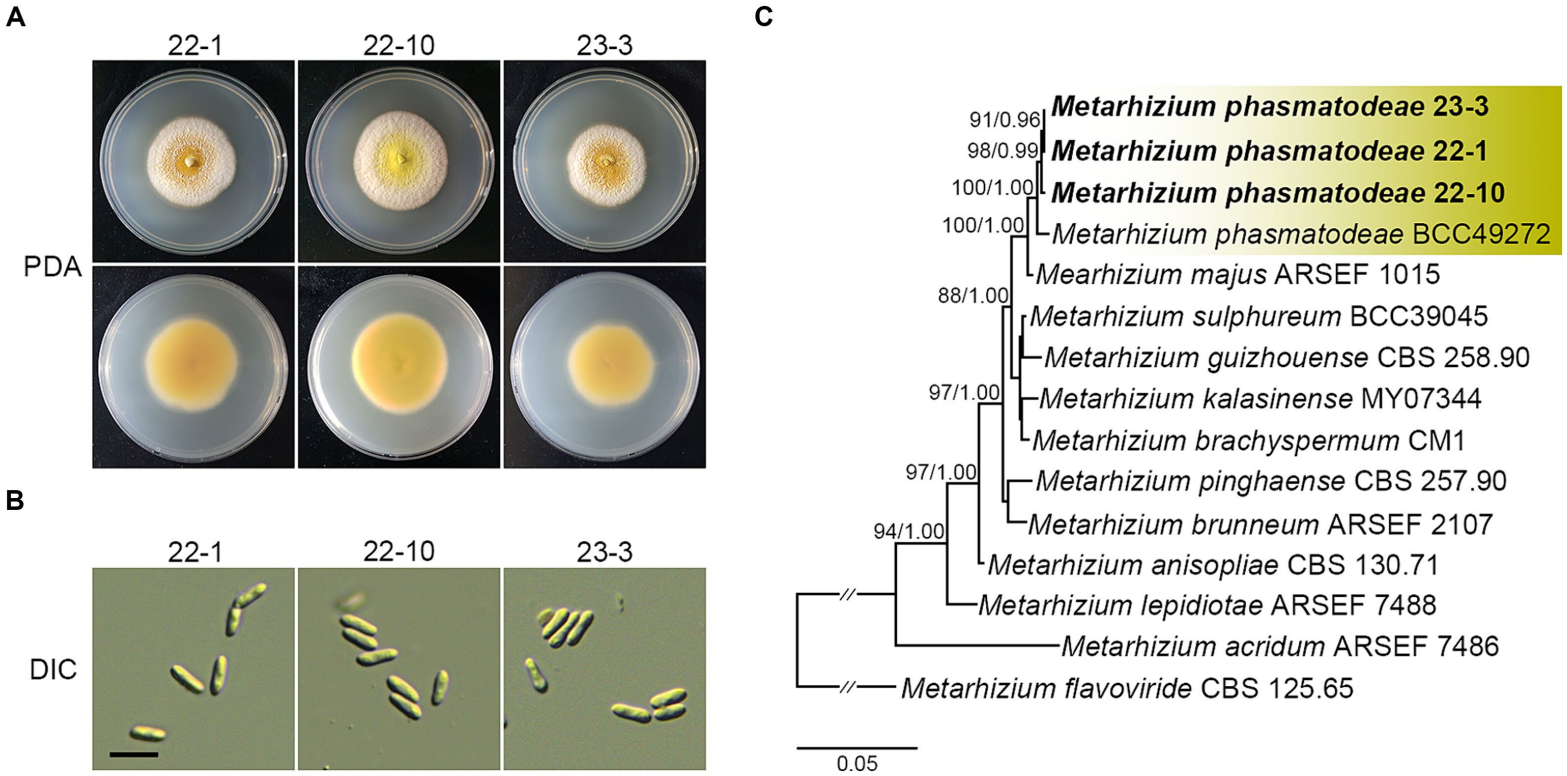
Morphological characterization and phylogenetic reconstruction of isolates in this study. **(A)** Colonies on PDA after 14 days. **(B)** Conidia on PDA. Scale bar =10 μm. **(C)** Phylogenetic reconstruction of *M. anisopliae* species complex based on ITS and 5′*tef* sequences using ML and Bayesian analysis. Number on the nodes are ML bootstrap / Bayesian posterior probability values above 70% (MLBS) or 0.7 (BPP).

As described above, a significant number of fungus-infested insects were collected during the summer season. Notably, these insects exhibited green spore masses covering their bodies, a distinctive trait indicative of *Metarhizium* species infection (Thanakitpipattana et al., 2020). Thus, we isolated fungal strains from the carcasses of *R. mikado* to clearly identify the *Metarhizium* strains infecting *R. mikado*. Diseased stick insects were placed on PDA to isolate pure fungal cultures, and three putative *Metarhizium* strains were successfully obtained. Then we analyzed the phenotypic characteristics of vegetative growth and asexual reproduction. Colonies of all strains showed yellow with a white edge and robust sporulation when grown on PDA (Figure 3A). Conidia produced on PDA were single-celled and cylindrical shape (Figure 3B). Furthermore, a multigene alignment was conducted using DNA barcode genes, including the internal transcribed spacer (ITS) and 5′ intron-rich region of the translation elongation factor 1-alpha (5’*tef*). All three strains exhibited high similarity to the barcode gene sequence of the *M. phasmatodeae* strain BCC49272, with ITS showing 99.56–99.71% similarity and 5’*tef* showing 98.54–98.78%. In addition, phylogenetic analyses employing both maximum likelihood (ML) and Bayesian methods further confirmed a close relationship between the strains in this study and the *M. phasmatodeae* strain (BCC49272). As previously reported, *M. phasmatodeae* is a recently identified species known to infect stick insects (Phasmatodea) (Mongkolsamrit et al., 2020; Thanakitpipattana et al., 2020). In summary, based on comprehensive morphological and taxonomic characterization, the three strains were conclusively identified as belonging to *M. phasmatodeae*, suggesting their potential as biocontrol agents against Phasmatodea insects (Figure 3C).

### 3.3 Insect bioassay

**Figure 4:**
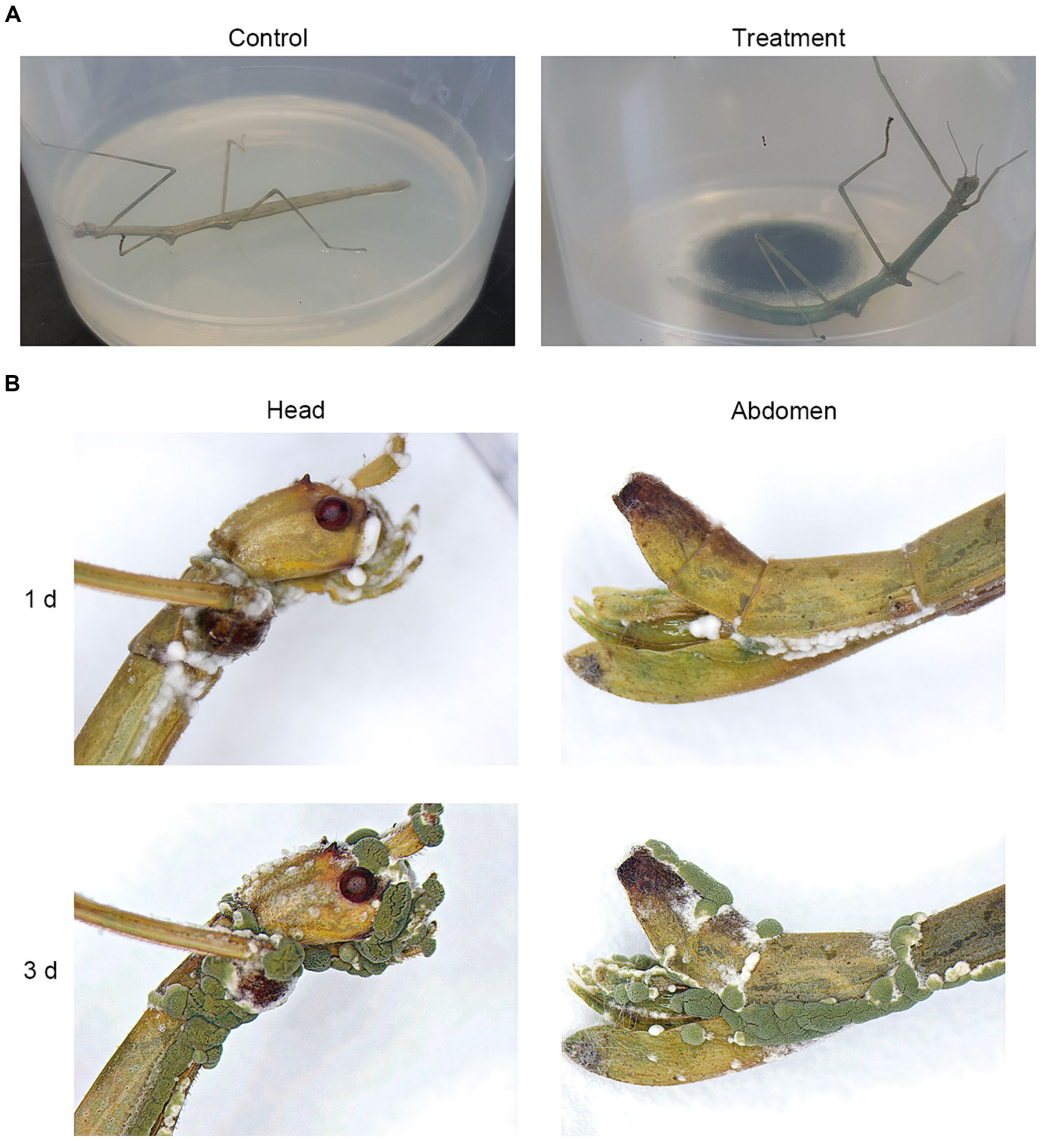
Biocontrol effects of *M. phasmatodeae* on *R. mikado*. **(A)** Each insect sample was exposed to the culture of *M. phasmatodeae*. Pure PDA culture was used as a control. The treated insect was transferred to a plastic dish and checked every day until 100% mortality was achieved. **(B)** Representative images of *R. mikado* after treatment with *M. phasmatodeae*. Dead insects were removed from the dishes and incubated for an additional indicated period (1 or 3 days) under moist conditions until mycelial outgrowth was observed.

To assess the pathogenicity of isolated *M. phasmatodeae* against *R. mikado*, healthy *R. mikado* adults were directly exposed to the 14-day-old fungal culture for 1 h and subsequently transferred to a clean dish (Figure 4A). Stick insects exposed to a clean PDA culture served as the control group. Following the treatments, all individuals in the experimental group succumbed within 6 days, in contrast to the control group, which survived throughout the experimental period. The external emergence of mycelia from the carcasses and the formation of green conidia were observed from the third day onward (Figure 4B). Taken together, our findings affirm that the *M. phasmatodeae* strains examined in this study exhibit biocontrol activity against *R. mikado*.

### 3.4 Association between *R. mikado* survival and environmental variables

**Figure 5:**
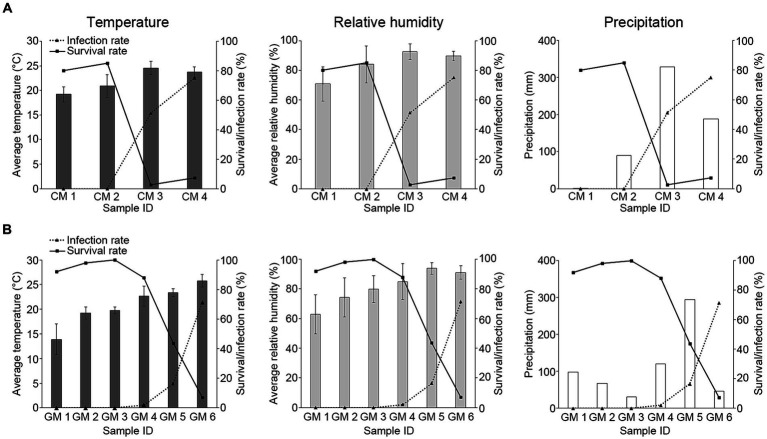
Investigation of the relationship between environmental factors and *M. phasmatodeae* infection against *R. mikado*. The values in the bar graph indicate various environmental factors in **(A)** 2022 and **(B)** 2023. Solid and dashed lines indicate survival and infection rates, respectively. Average temperature and Average relative humidity are represented as average values for 2 weeks derived from HOBO data loggers at each sampling site. Exceptionally, temperature and relative humidity values of GM1 were investigated using Automatic Weather Station (AWS) data. The values of precipitation represent the total accumulation for 2 weeks derived from the nearby AWS.

Given the observed mortality of *R. mikado* during the summer season, our inquiry focused on understanding the factors contributing to the heightened virulence of *M. phasmatodeae* in forest ecosystems. As described earlier, an increase in rainfall was noted during the summer seasons of both 2022 and 2023, resulting in hot and humid conditions. We collected data on average temperature and relative humidity using data loggers at the sampling sites, and accumulated precipitation data were obtained from AWS (Table 2). Subsequently, each environmental factor was compared with survival and infection rates (Figure 5).

The analysis revealed a decline in survival rates with increasing temperature and humidity, accompanied by a rapid increase in infection rates. This implies an inverse relationship between the infection rate and survival rate, indicating a positive association between the infection rate and environmental factors. Notably, the high infection rate on the last sampling dates in both 2022 and 2023 was observed during periods of low precipitation. In this period, the warm and humid conditions following previous rainfall during the rainy season may have led to elevated infection rates, even as precipitation declined. In summary, our findings suggest fluctuations in the survival of *R. mikado* were influenced by the rainy season, which in turn affected temperature and humidity conditions. Our observation aligns with previous studies indicating that the insecticidal performance of entomopathogenic fungi could be influenced by environmental conditions such as humidity and temperature (De La Rosa et al., 2000; Arthurs and Thomas, 2001; Athanassiou et al., 2017). Therefore, we aimed to analyze their correlation through further statistical analysis.

### 3.5 Validation of the *R. mikado* survival model

**Figure 6:**
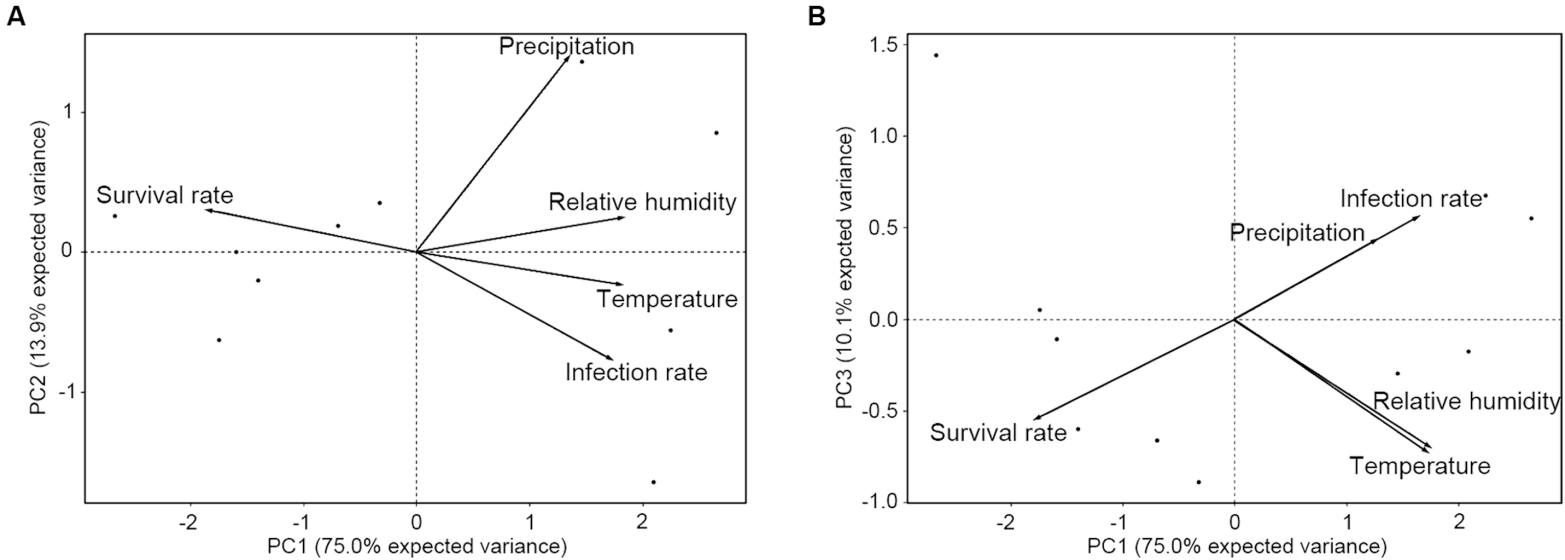
Principal component analysis (PCA) on the survival and infection rates of *R. mikado*, relative humidity, and temperature. Each biplot represents the combination of **(A)** PC1 and PC2, as well as **(B)** PC1 and PC3. The percentages of variance explained by axes or components (PC1-3) are shown in parentheses. Vectors indicate significant correlations between survival rate, infection rate, and environmental factors.

To validate the *R. mikado* survival model, principal component analysis (PCA) was employed to understand relationships among various environmental factors (Table 2; Supplementary Table S4). This analysis aimed to identify correlated variables and ascertain which factors contributed most significantly to the variation in stick insect survival (Figure 6). The cumulative proportion of explanation for the first three dimensions (PC1, PC2, and PC3) was 75.0, 13.9, and 10.0%, respectively, with a total of 98.9% of the variances explaining the model. PC1 and PC2 with an eigen values of 3.75 and 0.69 revealed a notable negative correlation between *R. mikado* survival and *M. phasmatodeae* infection, while precipitation exhibited a somewhat weaker correlation with the survival rate and infection rate (Figure 6A). Conversely, PC1 and PC3 with an eigen values of 3.75 and 0.50 indicated a positive correlation between precipitation and the infection rate, with both factors negatively correlated with the survival rate (Figure 6B). To summarize, the survival rate displayed a significant negative correlation with infection rate, temperature, relative humidity, and precipitation. However, using the Spearman correlation coefficient, no statistically significant correlation was observed between precipitation and the infection rate, as well as precipitation and temperature (*p* > 0.05) (Supplementary Figure S1).

**Figure 7:**
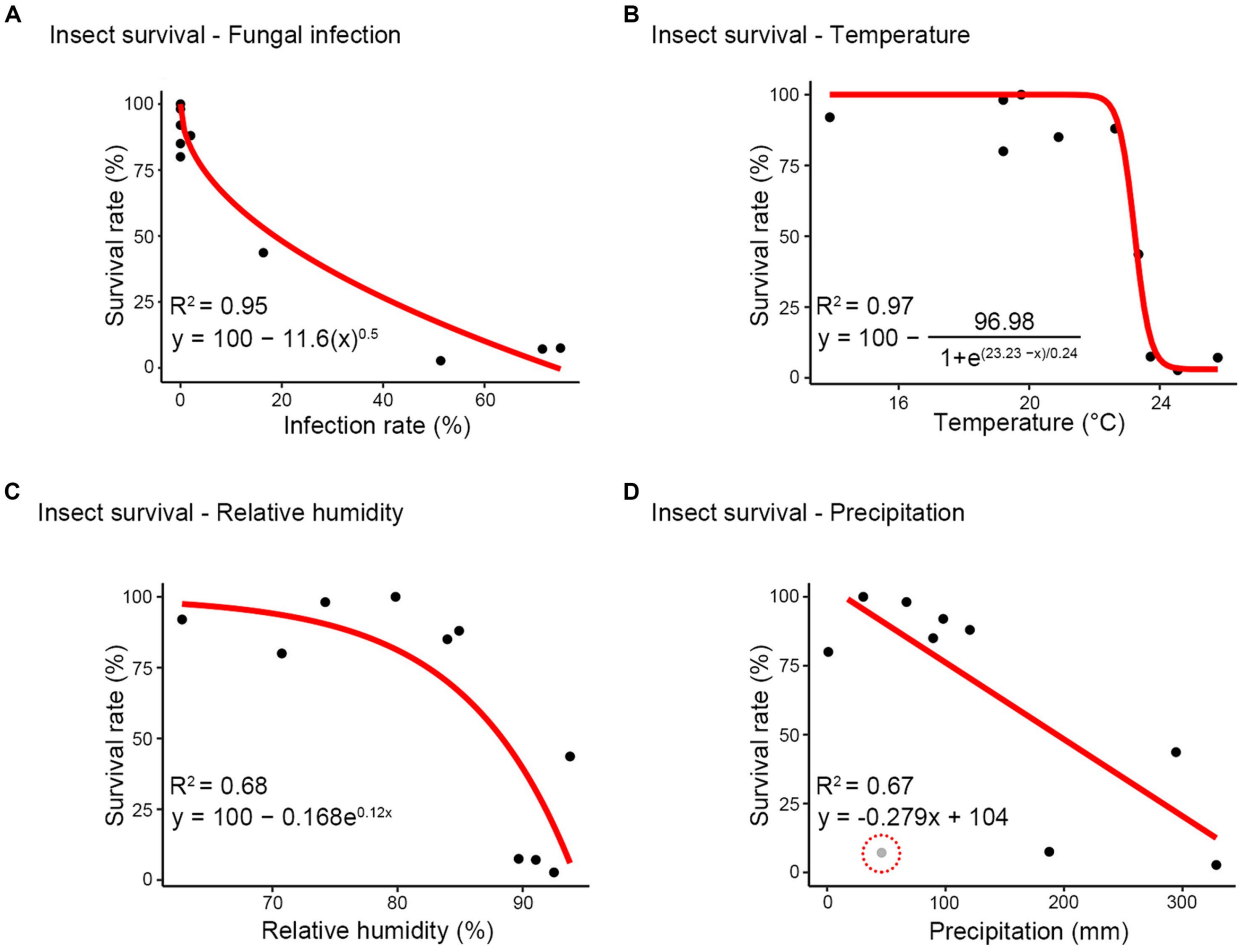
Modeling between survival rate and other factors. **(A)** infection rate; **(B)** temperature; **(C)** relative humidity; **(D)** precipitation. Red line shows the relationship and the equation of each red line is described beside the red line. The performance of each model was examined using the coefficient of determination (*R*^2^). Gray dot (highlighted using red dash line) excluded when creating the model.

Further, considering the close relationship observed between insect survival and each variable (infection rate, temperature, relative humidity, and precipitation), we investigated whether these associations could be proposed as a host-pathogen interaction model. A significant curvilinear or linear relationship was identified between survival rate and infection rate (R^2^ = 0.95), temperature (R^2^ = 0.97), relative humidity (R^2^ = 0.68), and precipitation (R^2^ = 0.67) (Figure 7). This relationship exhibited a rapid decline in survival rate with low infection rates, followed by a gradual decrease as infection rates increased (Figure 7A). Temperatures ranging from 14°C to 23°C had minimal impact on the survival rate, while beyond 23°C, the survival rate decreased to less than 0.5 (Figure 7B). Additionally, as relative humidity increased, there was a progressively sharp decline in the survival rate, dropping below 50% at humidity levels exceeding approximately 85% (Figure 7C). The relationship between precipitation and survival rate showed a negative linear correlation (Figure 7D). Overall, these results suggest that the survival of *R. mikado* exhibits a nonlinear/linear relationship with various factors, including fungal infection, temperature, relative humidity, and precipitation.

## 4 Discussion

The order Phasmatodea, encompassing stick insects, is known for harboring significant phytophagous pests affecting agriculture and forestry in various countries (Campbell, 1960; Prathapan et al., 2008). In the Republic of Korea, five species of insects, namely *R. mikado*, *R. koreanus*, *Micadina phluctainoides*, *M. yasumatsui*, and *Phraortes illepidus*, belong to Phasmatodea. Among these, *R. mikado* is recognized as a forest insect pest that causes severe defoliation by feeding on leaves from diverse deciduous trees (Jung et al., 2020).

To manage stick insects, a common approach involves the use of commercial sticky traps, which are encased around the tree trunks to capture hatched young nymphs climbing up from the ground (Korean Forest Service, 2023). Additionally, chemical control methods are employed, utilizing synthetic pesticides such as fenitrothion and ethofenprox. However, the application of sticky traps comes with inherent challenges, requiring individual installation in the forest and incurring substantial labor costs. Similar to chemical control methods, sticky traps also pose the disadvantage of impacting non-target organisms (Blacquière et al., 2012; Rahman et al., 2020). Moreover, the efficacy of chemical control may diminish over time due to the development of resistance in target insects (Hemingway and Ranson, 2000).

In this study, *M. phasmatodeae*, first reported in Korea and previously documented in Thailand and China, has demonstrated pathogenicity for stick insects as hosts (Thanakitpipattana et al., 2020; Zhao et al., 2023), suggesting its potential as a promising biocontrol agent. Our observation revealed that all insects inoculated with *M. phasmatodeae* succumbed within 5 days. Considering the rapid onset, *M. phasmatodeae* holds promise as a biocontrol agent against stick insect outbreaks in forest ecosystems (Sabbahi et al., 2022; Vivekanandhan et al., 2022; Perumal et al., 2023). However, for application in forest ecosystems, additional host specificity tests are necessary. To enhance the effectiveness of fungal infection, it is essential to develop spray techniques that consider the ideal timing in accordance with environmental conditions.

We established the correlations among environmental factors, insect survival, and fungal infection through various statistical analyses. PCA indicated a minimal correlation between precipitation and infection rate in PC1 and PC2, while a positive correlation was identified between precipitation and infection rates in PC1 and PC3. This disparity can be attributed to the characteristics of the rainy season, where precipitation undergoes rapid fluctuations, experiencing sudden increases followed by subsequent decreases (Wang et al., 2007). These changes, driven by the hot and humid conditions during the rainy season, persist even with reduced rainfall, enhancing the insecticidal performance of *M. phasmatodeae*. Consequently, the infection rate peaked on the last sampling date for each year, despite decreased precipitation, influencing the outcomes of PCA. Furthermore, modeling was conducted to examine the survival rates and various other factors. According to the infection rate-survival rate formula, a 16% or higher infection rate results in a 50% mortality rate among stick insects. Therefore, even a relatively low infection rate could induce a significant number of deaths in the stick insect.

The extensive mortality observed in *R. mikado* may be influenced by the population density of stick insects. A similar study conducted on locust populations reported a low infection rate (Shah et al., 1998), suggesting that population density could play a role in this disparity. The sampling sites in this study were forests experiencing significant stick insect outbreaks, resulting in much higher population densities than usual. The heightened density of hosts facilitates the acceleration of horizontal transmission among individuals (Roy and Pell, 2000). Additionally, the collective deaths of the stick insects could be related to the reproduction strategy. The formation of colonies through asexual parthenogenesis may indicate relatively equal susceptibility to a fungus (Steinkraus, 2006).

In conclusion, this study confirmed the natural infection of *R. mikado* by *M. phasmatodeae* and observed the mass mortality phenomenon in the stick insects due to the increased virulence of *M. phasmatodeae* in the hot and humid environment formed during the rainy season. Our findings will contribute to predicting population levels for the next year based on the rainy season. The adult female stick insects normally lay eggs per day in the summer (Kobayashi et al., 2014; Lee et al., 2018). This trait implies that the total number of eggs laid may vary depending on the onset of the rainy season, consequently impacting population levels in the subsequent year. The fecundity of insects is one of the crucial factors in predicting the size of the next population (Peters and Barbosa, 1977; Powell and Bentz, 2009). Forecasting the future population could potentially assist in formulating pest control strategies. To ascertain whether this phenomenon is exclusive to *R. mikado* or common among stick insects within the Phasmatodea order, further research will be necessary.

## Data availability statement

The datasets presented in this study can be found in online repositories. The names of the repository/repositories and accession number(s) can be found in the article/Supplementary material.

## Author contributions

DM: Data curation, Formal analysis, Investigation, Methodology, Validation, Visualization, Writing – original draft, Writing – review & editing. SS: Formal analysis, Investigation, Software, Validation, Visualization, Writing – original draft, Writing – review & editing. N-HL: Data curation, Methodology, Software, Writing – original draft. MB: Funding acquisition, Resources, Writing – original draft. S-JP: Funding acquisition, Resources, Writing – original draft. K-HK: Data curation, Methodology, Software, Writing – original draft. HS: Project administration, Supervision, Writing – original draft, Writing – review & editing. J-KJ: Conceptualization, Funding acquisition, Investigation, Project administration, Supervision, Writing – original draft, Writing – review & editing.

## Footnotes

^1^ https://species.nibr.go.kr/index.do
