# Supplementary material for: Survival dynamics of stick insect and the impact of environmental factors on natural fungal infection during the rainy season
Source: Front Microbiol. 2024 Apr 24;15:1383055. doi: 10.3389/fmicb.2024.1383055 (PMC11076836; doi:10.3389/fmicb.2024.1383055)
Supplement: Supplementary file 1 [file Data_Sheet_1.docx]

**Supplementary information**

**Survival dynamics of stick insect and the impact of environmental factors on natural fungal infection during the rainy season**

**Donggyu Min^1,2^**^†^**, Soobin Shin^1^**^†^**, Noh-Hyun Lee^1^, Min Jeong Baek^3^, Sun-Jae Park^3^, Kwang-Hyung Kim^1,4^, Hokyoung Son^1,4*^, and Jong-Kook Jung^2*^**

^1^Department of Agricultural Biotechnology, Seoul National University, Seoul, Republic of Korea

^2^Department of Forest Environment Protection, Kangwon National University, Chuncheon Republic of Korea

^3^National Institute of Biological Resources, Incheon, Republic of Korea

^4^Research Institute of Agriculture and Life Sciences, Seoul National University, Seoul, Republic of Korea

†These authors contributed equally to this work and share first authorship.

* Correspondence:

Hokyoung Son: hogongi7@snu.ac.kr

Jong-Kook Jung: jkjung@kangwon.ac.kr

Keywords: *Ramulus mikado*, *Metarhizium phasmatodeae*, Rainy season, Biocontrol agent, Entomopathogenic fungi

**Figure S1 Correlation analysis between variables.** Red indicates a positive correlation, while blue indicates a negative correlation. Each number represents the correlation coefficient, and only those with a *p*-value of 0.05 or less are highlighted with a white line. The analysis was performed using Spearman correlation.

**
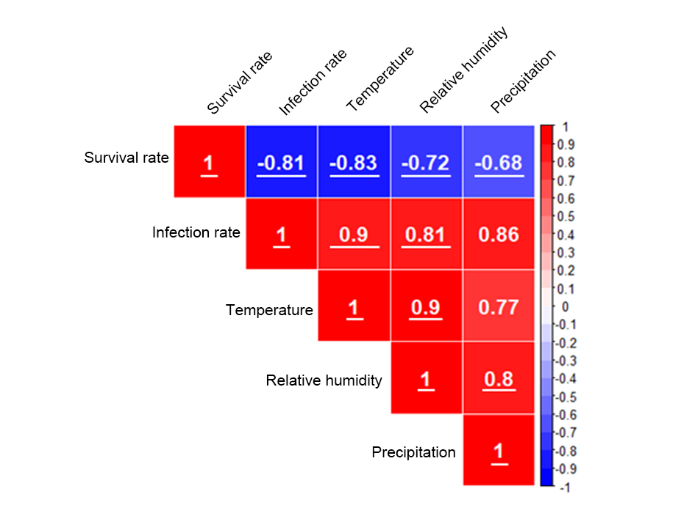
**

**Table S1 Primers used in this study.**

| **Barcode region** | **Primer** | **Sequence (5’ → 3’)** |
| --- | --- | --- |
| 5’tef | EF1T | ATGGGTAAGGARGACAAGAC |
|  | EF2T | GGAAGTACCAGTGATCATGTT |
| ITS | ITS1 | GARTGYCCDGGDCAYTTYGG |
|  | ITS4 | CCNGCDATNTCRTTRTCCATRTA |

**Table S2 Previously unrecorded species with details of GenBank accession numbers of the sequences.**

| **Species** | **Strain name** | **GenBank accession number** | |
| --- | --- | --- | --- |
|  |  | **ITS** | **5’ tef** |
| Metarhizium phasmatodeae | 22-1 | PP204037 | PP212873 |
|  | 22-10 | PP204038 | PP212874 |
|  | 23-3 | PP204039 | PP212875 |

**Table S3 Kaplan-Meier Log-rank pairwise comparisons of survival curves.** X^2^ represents the value of chi-square, and *p*-values lower than 0.001 were expressed as *p* < 0.001.

| **2022** | **CM1** | **CM2** | **CM3** | **-** | **-** |
| --- | --- | --- | --- | --- | --- |
| **CM2** | X^2^ = 0.171 | **-** | **-** | **-** | **-** |
|  | p = 0.679 |  |  |  |  |
| **CM3** | X^2^ = 54.969 | X^2^ = 54.105 | **-** | **-** | **-** |
|  | p < 0.001 | p < 0.001 |  |  |  |
| **CM4** | X^2^ = 48.547 | X^2^ = 52.462 | X^2^ = 1.195 | **-** | **-** |
|  | p < 0.001 | p < 0.001 | p = 0.274 |  |  |
| **2023** | **GM1** | **GM2** | **GM3** | **GM4** | **GM5** |
| **GM2** | X^2^ = 2.110 | - | - | - | - |
|  | p = 0.146 |  |  |  |  |
| **GM3** | X^2^ = 4.125 | X^2^ = 0.943 | - | - | - |
|  | p = 0.042 | p = 0.331 |  |  |  |
| **GM4** | X^2^ = 0.342 | X^2^ = 4.045 | X^2^ = 6.320 | - | - |
|  | p = 0.556 | p = 0.044 | p = 0.012 |  |  |
| **GM5** | X^2^ = 25.643 | X^2^ = 38.329 | X^2^ = 39.914 | X^2^ = 22.831 | - |
|  | p < 0.001 | p < 0.001 | p < 0.001 | p < 0.001 |  |
| **GM6** | X^2^ = 81.694 | X^2^ = 101.807 | X^2^ = 99.886 | X^2^ = 86.046 | X^2^ = 34.758 |
|  | p < 0.001 | p < 0.001 | p < 0.001 | p < 0.001 | p < 0.001 |

**Table S4 Sample factor loading matrix of principle components.**

| **Parameters** | **PC1** | **PC2** | **PC3** | **PC4** | **PC5** |
| --- | --- | --- | --- | --- | --- |
| Survival rate | -0.4642 | -0.3685 | 0.2300 | 0.7082 | -0.3071 |
| Infection rate | 0.4505 | 0.4239 | -0.4346 | 0.6545 | -0.0058 |
| Temperature | 0.4246 | -0.5805 | -0.3560 | -0.1577 | -0.5754 |
| Relative humidity | 0.4384 | -0.5309 | 0.2471 | 0.2119 | 0.6481 |
| Precipitation | 0.4573 | 0.2562 | 0.7553 | 0.0173 | -0.3931 |
| **Eigenvalue** | 3.7510 | 0.6954 | 0.5039 | 0.0348 | 0.0150 |
| **Variance (%)** | 0.7502 | 0.1391 | 0.1008 | 0.0070 | 0.0030 |
| **Cumulative Variance (%)** | 0.7502 | 0.8893 | 0.9900 | 0.9970 | 1.0000 |
